# The Human Positive Cofactor 4 is a Promising Chemotherapeutic Target in Lung Adenocarcinoma

**DOI:** 10.1155/2021/9958483

**Published:** 2021-12-03

**Authors:** Tianyu Sun, Jingge Zhang, Xiaoqing Fan, Tan Long, Shaolin Tao, Poming Kang, Qunyou Tan

**Affiliations:** Department of Thoracic Surgery, Daping Hospital, Army Medical University, Chongqing 400042, China

## Abstract

Reduced sensitivity to chemotherapeutic drugs is almost inevitable in lung adenocarcinoma patients. Thus, understanding the relevant mechanisms is urgent. Positive cofactor 4 (PC4) was at first revealed to be a coactivator of basal transcription. Previous research has shown that PC4 participates in various cellular processes in normal and malignant cells. However, it is still unknown whether PC4 participates in altering the lung adenocarcinoma cell sensitivity to chemotherapy, and the relevant mechanisms remain to be explained. In this study, we discovered that PC4 was overexpressed in cisplatin-resistant lung adenocarcinoma cells. PC4 decreased cisplatin's cytotoxic effects on lung adenocarcinoma *in vivo* and *in vitro*. Furthermore, PC4 positively correlated with SOX9 in multiple cancers. PC4 was an upstream regulator of SOX9 in lung adenocarcinoma. Furthermore, PC4 mediated lung adenocarcinoma cell sensitivity to the HIF-PH inhibitor DMOG and the mTOR inhibitor rapamycin, and PC4 mediated the synergistic effect of DMOG and cisplatin. Finally, PC4 destabilized HIF-1*α* upon cisplatin treatment. Our research showed that PC4 participates in mediating lung adenocarcinoma cell sensitivity to multiple drugs. Mechanistically, PC4 governs multiple downstream pathways associated with chemotherapy resistance, including the SOX9 and HIF-1*α* pathways. Thus, PC4 is a promising chemotherapeutic target in lung adenocarcinoma.

## 1. Introduction

Reduced sensitivity to chemotherapeutic drugs is almost inevitable in lung adenocarcinoma (LUAD) therapy. Recently, many genes were discovered as targets to predict the progression or therapeutic response of lung adenocarcinoma [1]. Although many new therapeutic targets have been discovered and targeted drugs have been developed, most patients eventually develop resistance to these targeted drugs and ultimately have a poor prognosis [2–5]. Thus, improving lung adenocarcinoma cell sensitivity to chemotherapy is still of great clinical significance.

Positive cofactor 4 (PC4), or SUB1, was at first discovered to be a coactivator of the basal transcription [6, 7]. According to previous reports, PC4 is localized to the nucleus and facilitates activator-dependent transcription [8, 9]. Previous research shows that PC4 participates in a variety of cellular processes, such as DNA repair, DNA replication, transcription, and chromatin organization [6, 9–12]. PC4 is also reported to participate in multiple cancer progressions. PC4 promotes tumor proliferation and metastasis in breast cancer [13]. Inhibiting PC4 suppresses the tumorigenesis and lung metastasis of osteosarcoma [14]. Our previous research shows that inhibiting PC4 reduces the lymphatic metastasis of lung adenocarcinoma [8]. However, the role of PC4 in mediating lung adenocarcinoma cell sensitivity to chemotherapy remains to be explored, and the relevant mechanisms remain to be further elucidated.

SRY-box transcription factor 9 (SOX9) is a SOX transcription factor and is located on chromosome 17 [15, 16]. SOX9 determines cell fate through downstream genes involved in maintaining pluripotency, directing cell lineage differentiation, and sustaining adult tissue homeostasis [17, 18]. Recent studies indicate a role of SOX9 in altering the progression and drug sensitivity of several types of tumors, including lung adenocarcinoma [19]. SOX9 promotes tumorigenesis in lung adenocarcinoma through transcriptionally regulating forkhead box A1 (FOXA1) [20]. In lung cancer, SOX9 promotes cancer cells resistant to cisplatin by increasing aldehyde dehydrogenase (ALDH) activity via ALDH1A1 [21].

In this study, PC4 is demonstrated to promote cell migration in lung adenocarcinoma cells without affecting cell growth and apoptosis. PC4 decreases lung adenocarcinoma sensitivity to cisplatin *in vivo* and *in vitro*. Mechanically, SOX9 is a downstream factor of PC4. PC4 overexpression upregulates while PC4 knockdown inhibits the expression of SOX9. PC4 also decreases lung adenocarcinoma cell sensitivity to the HIF-PH inhibitor DMOG and the mTOR inhibitor rapamycin. DMOG exerts a synergistic enhancive effect on cisplatin, which is weakened by PC4. Moreover, PC4-decreased cell sensitivity to cisplatin is accompanied by destabilization of HIF-1*α*.

## 2. Methods

### 2.1. Cell Culture

HEK293 T cells and human lung adenocarcinoma cell lines (H1299 and PC-9) were obtained and maintained in our laboratory as previously described [22]. The medium of RPMI and DMEM medium (HyClone, USA) and fetal bovine serum (FBS) (Gibco, USA) were used to culture the cells.

### 2.2. Reagents

Cisplatin (MCE, USA) was freshly weighed and dissolved in PBS. A-485, DMOG, LY294002, MG132, MHY1485, and rapamycin (MCE, USA) were dissolved in DMSO.

### 2.3. Plasmids

The pLVX-shRNA2 and pLVX-IRES-ZsGreen1 plasmids (Clontech, USA) were selected as the lentiviral vectors for PC4 knockdown and overexpression, respectively. The target sequences for PC4 knockdown were PC4 sh1 GAAGGAACAGATTTCTGACAT; PC4 sh2 GACAGGTGAGACTTCGAGA; and PC4 sh3 GCAAAGTGCTAATTGATAT. The PC4-knockdown negative control (PC4 NC) sequence was designed online from the Invivogen website and was GCTGGATACGGATAATTAACA. The sequences were cloned into pLVX-shRNA2 in accordance with previous research [23]. The pLVX-IRES-zsGreen1 vector containing SUB1 mRNA CDSs was used for the PC4-overexpression group (PC4), and the empty pLVX-IRES-zsGreen1 was adopted as the vector group (vec). The SOX9-overexpression plasmids (Genechem, China) were transfected and screened with puromycin.

### 2.4. Lentivirus Production

The production of PC4-knockdown and PC4-overexpression lentivirus was conducted by cotransfecting HEK293 T cells with the pLVX-shRNA2 vector (or pLVX-IRES-ZsGreen1), the psPAX2, and the pMD2.G (Addgene, USA) plasmids as previously described [22, 23]. 48 hours after transfection, virus particles were harvested.

### 2.5. Cell Viability

The CCK-8 test was accomplished using the Cell Counting Kit-8 (CCK-8) (Bioss, China) as we previously described [22]. The OD values were respectively obtained in the blank control, control, and experimental group (OD_Blank_, OD_Control_, OD_Experiment_). The cell viability was calculated as previously described [22]. The relative survival ratio was calculated as follows: cell viability _combinational therapy_/cell viability _cisplatin_ × 100%. The enhancive effect of drug on cisplatin is calculated as follows: (1-cell viability _combinational therapy_/cell viability _cisplatin_) × 100%. Each result is representative of 6 replicate samples.

### 2.6. Cell Apoptosis Analysis

The Annexin V-FITC/PI assay was applied to evaluate cell apoptosis. Cells were seeded in advance in a 6-well plate. 48 hours later, drugs were administered. Cell apoptosis was analyzed 24 hours after drug administration. The flow cytometer (Beckman Coulter, USA) was used to detect cell apoptosis through the Annexin V-FITC/PI apoptosis kit (KeyGEN BioTECH, China).

### 2.7. Western Blot

RIPA lysis containing protease inhibitor (Thermo Scientific, USA) was used for protein extractions. Equal proteins were loaded for SDS-PAGE and blotted as reported by previous research [24]. The antibodies used were PC4 (ab72132, Abcam), GAPDH (#2118, CST), caspase 3 (#14220, CST), cleaved caspase 3 (#9664, CST), PARP (#9542, CST), and HIF-1*α* (bs-0737R, Bioss).

### 2.8. Cell Migration Assay

Transwell assay was used to evaluate the cell migration. About 3 × 10^4^ lung adenocarcinoma cells were suspended in 200 *µ*l non-FBS RPMI and planted in the upper chamber (Millicell, USA). 600 *µ*l RPMI including 20% FBS was placed in the lower chamber. After 24-hour incubation, the cells underwent paraformaldehyde fixing and crystal violet staining. Then microscopic quantification was performed for data analysis. The results represent three independent experiments.

### 2.9. Immunofluorescence Assay

Cells were seeded in 96-well plates for 24 hours, followed by wash with PBS and fixation with 4% formaldehyde. BSA (5%) containing 0.3% Triton was used to block the nonspecific proteins, and SOX9 primary antibodies (Bioss, China) were added at a dilution of 1 : 100 and incubated overnight. The secondary antibody tagged with Alexa Fluor 546 (Thermo Fisher, USA) was added and incubated for 1 hour. DAPI (Beyotime, China) was used to counterstain the nuclei. A Nikon Eclipse Ti fluorescence microscope was applied to capture the images.

### 2.10. Tumor Xenograft Model

Six-week nude mice were obtained from Daping Hospital Laboratory Animal Center. The nude mice underwent subcutaneous implantation of H1299 vec and H1299 PC4 cells. The cisplatin treatment and tumor size calculation were in accordance with our previous research [22]. The animal research was conducted under the approval of the Animal Care and Use Committees of Daping Hospital.

### 2.11. Statistical Analysis

The SPSS 18.0 (SPSS Inc., USA) software was applied to finish the statistical analyses. The data was considered significant when the *p* value was less than 0.05. The GraphPad Prism 5 (GraphPad Software Inc., USA) software was applied to produce the figures.

## 3. Results

### 3.1. PC4 Is Overexpressed in Cisplatin-Resistant Cancer Cells

**Figure 1:**
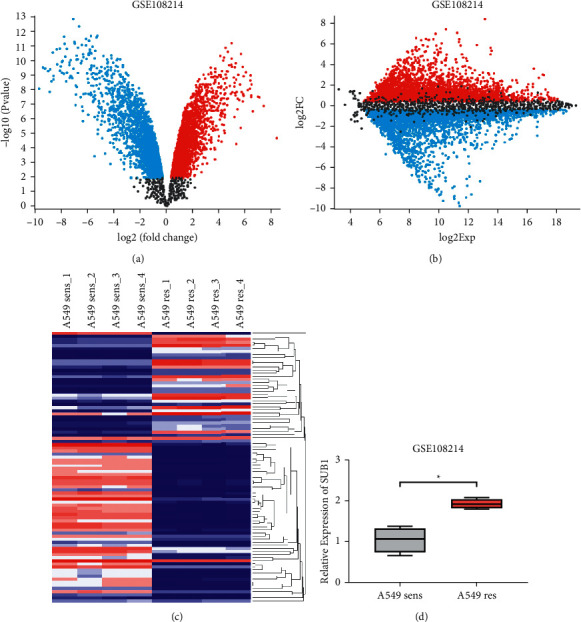
PC4 is overexpressed in cisplatin-resistant lung adenocarcinoma cells. The volcano plot (a) and the mean-difference plot (b) show the differentially expressed genes of GSE108214. (c) Heatmap showing some of the differentially expressed genes in GSE108214. (d) The relative expression of SUB1 in CDDP-sensitive A549 (A549 sens) and CDDP-resistant A549 (A549 res) cells in GSE108214. ^*∗*^*p* < 0.05.

First, we searched the GEO database for bioinformatic data on cisplatin resistance in lung cancer. The GSE108214 series was analyzed. GSM2892607, GSM2892612, GSM2892615, and GSM2892622 were included in the A549 cisplatin-sensitive group (A549 sens). GSM2892609, GSM2892614, GSM2892617, and GSM2892623 were included in the A549 cisplatin-resistant group (A549 res).  Figure 1(a) shows the volcano plot, and Figure 1(b) shows the mean-difference plot of the data.  Figure 1(c) shows the heatmap of some of the differentially expressed genes. The expression of the SUB1 gene was analyzed in the database. As demonstrated in Figure 1(d), SUB1 mRNA expression was elevated in the A549 res group in comparison with the A549 sens group (LogFC = 0.947, *p* = 0.002).

### 3.2. The Impact of PC4 on the Malignant Phenotypes of Lung Adenocarcinoma Cells

**Figure 2:**
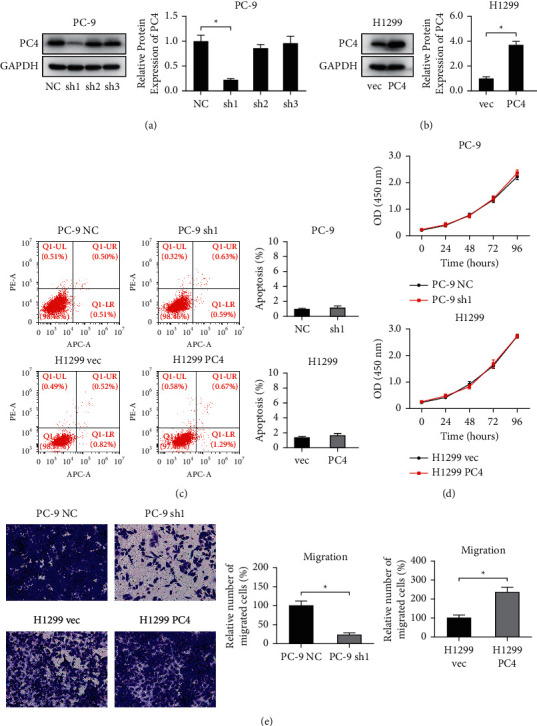
The impact of PC4 on the malignant phenotypes of lung adenocarcinoma cells. (a), (b) Western blot assay revealed the protein levels of PC4 after LUAD cells were infected with PC4-knockdown or PC4-overexpression lentivirus. (c) The apoptosis of PC4-knockdown or PC4-overexpression LUAD cells was evaluated by flow cytometry. (d) The growth of PC4-knockdown or PC4-overexpression LUAD cells was analyzed via CCK-8 experiments. (e) The migration of LUAD cells following PC4 knockdown or overexpression was detected via Transwell assay. ^*∗*^*p* < 0.05.

First, PC4 was knocked down in PC-9 cells and overexpressed in H1299 cells through lentivirus infection (Figures 2(a) and 2(b)). As shown in Figure 2(a), sh1 showed a superior inhibitory effect on the expression of PC4 and was selected for further research. Next, we assessed the impact of PC4 on the malignant phenotypes in lung adenocarcinoma. As shown in Figure 2(c), flow cytometry showed that the apoptosis levels in the PC4-knockdown PC-9 cells (1.01 ± 0.36% vs. 1.22 ± 0.54%, *p* > 0.05) and the PC4-overexpressing H1299 cells (1.34 ± 0.72% vs. 1.96 ± 0.61%, *p* > 0.05) were not statistically significant. Furthermore, CCK-8 assays demonstrated no significant changes in cell growth in the PC-9 sh1 cells in comparison with the PC-9 NC cells (Figure 2(d)). Similarly, the growth of the H1299 PC4 cells was not significantly different from that of the H1299 vec cells (Figure 2(d)). Interestingly, Transwell assays (Figure 2(e)) showed that the cell migration ability was significantly reduced in the PC-9 sh1 cells in contrast to the PC-9 NC cells (*p* < 0.001) and significantly increased in the H1299 PC4 cells in contrast to the H1299 vec cells (*p* < 0.001).

### 3.3. PC4 Decreases Cisplatin's Cytotoxic Effects on Lung Adenocarcinoma Cells Both *In Vivo* and *In Vitro*

**Figure 3:**
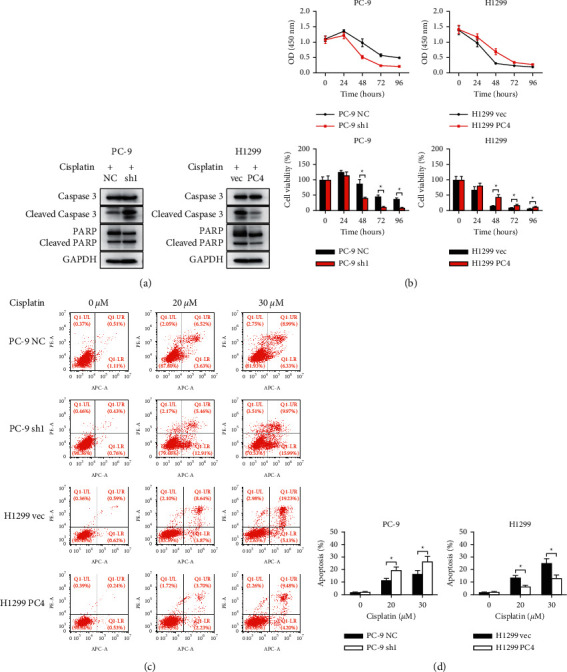
PC4 decreases cisplatin's cytotoxic effects on lung adenocarcinoma cells *in vitro*. (a) PC4-knockdown or PC4-overexpression LUAD cells underwent 24-hour treatment with 20 *μ*M cisplatin. The expression of apoptotic proteins was evaluated. (b) PC4-knockdown or PC4-overexpression LUAD cells were administrated with 20 *µ*M cisplatin for 96 hours. The CCK-8 assay was performed every 24 hours. (c), (d) PC4-knockdown or PC4-overexpression LUAD cells underwent 24-hour treatment with 0, 20, and 30 *µ*M cisplatin. Cell apoptosis was measured and analyzed by flow cytometry. ^*∗*^*p* < 0.05.

Next, we assessed the impact of PC4 on cell sensitivity to cisplatin. First, the levels of apoptotic proteins in PC4-knockdown and PC-overexpression cells treated with 20 *μ*M cisplatin for 24 hours were measured by western blot. According to the results, the caspase 3 cleavage and PARP cleavage were obviously activated in the PC-9 sh1 cells and obviously inhibited in the H1299 PC4 cells compared to their respective controls (Figure 3(a)). According to the CCK-8 assay, there was no statistical significance in the cell viability between the PC-9 sh1 cells and the PC-9 NC cells at 24 hours after cisplatin treatment (125.31 ± 5.16% vs. 114.81 ± 10.83%, *p* = 0.058). But the cell viability was significantly reduced in the PC-9 sh1 cells in comparison with the PC-9 NC cells at 48, 72, and 96 hours after cisplatin treatment (87.09 ± 13.15% vs. 41.11 ± 2.06% at 48 hours, 45.60 ± 4.62% vs. 12.00 ± 1.39% at 72 hours, 37.11 ± 3.39% vs. 9.13 ± 1.17% at 96 hours, *p* < 0.05) (Figure 3(b)). Similarly, cell viability was increased in the H1299 PC4 cells in comparison with the H1299 vec cells at 48, 72, and 96 hours after cisplatin treatment (14.99 ± 1.47% vs. 44.02 ± 7.60% at 48 hours, 9.13 ± 0.56% vs. 17.16 ± 3.05% at 72 hours, 5.76 ± 0.74% vs. 11.93 ± 1.24% at 96 hours, *p* < 0.05) (Figure 3(b)). Next, flow cytometry was applied to evaluate the apoptosis of PC4-knockdown and PC4-overexpression cells treated with 0, 20, or 30 *μ*M cisplatin for 24 hours. As demonstrated in Figures 3(c) and 3(d), there was no statistical significance in cell apoptosis between the untreated PC-9 NC and the PC-9 sh1 cells (1.83 ± 0.32% vs. 2.12 ± 0.24%, *p* > 0.05), but cisplatin-induced apoptosis significantly increased cell apoptosis in the PC-9 sh1 cells in comparison with the PC-9 NC cells (11.42 ± 1.46% vs. 19.50 ± 2.54% at 20 *μ*M cisplatin, 16.49 ± 2.64% vs. 26.54 ± 4.07% at 30 *μ*M cisplatin, *p* < 0.05). Similarly, cell apoptosis was not significantly different between the untreated H1299 vec cells and H1299 PC4 cells (1.37 ± 0.27% vs. 0.89 ± 0.35%, *p* > 0.05) but was obviously reduced in the H1299 PC4 cells treated with cisplatin than in the H1299 vec cells (13.51 ± 1.69% vs. 6.42 ± 0.89% at 20 *μ*M cisplatin, 25.14 ± 3.51% vs. 13.17 ± 2.47% at 30 *μ*M cisplatin, *p* < 0.05).

**Figure 4:**
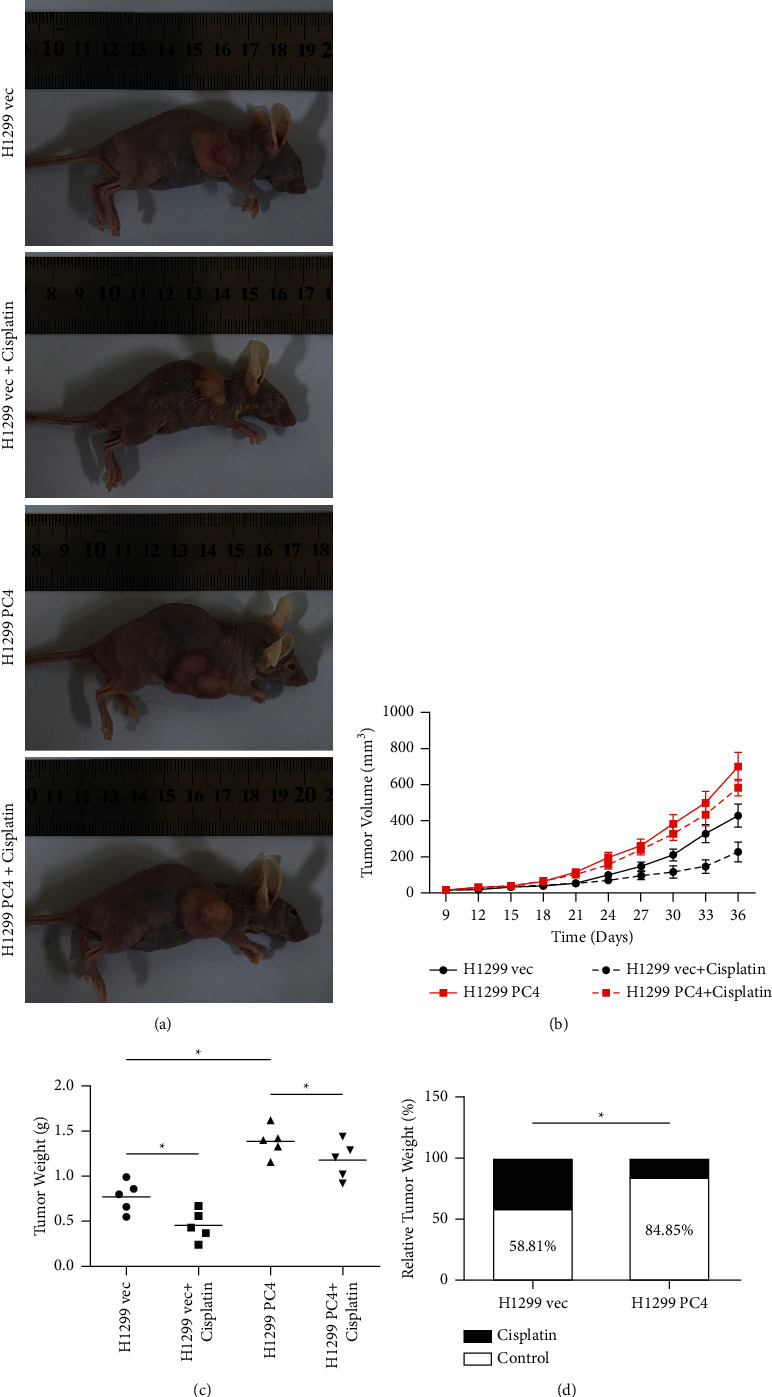
PC4 decreases lung adenocarcinoma sensitivity to cisplatin *in vivo*. H1299 vec and H1299 PC4 cells were subcutaneously injected into nude mice. Three mg/kg cisplatin was administered every 3 days. (a) The general image of tumor size. (b) The growth curves of tumors. (c) The weights of tumors in all groups. (d) Relative tumor weight was calculated to demonstrate the impact of PC4 on cisplatin sensitivity. ^*∗*^*p* < 0.05.

*In vivo* research was conducted through subcutaneous injection of H1299 vec and H1299 PC4 cells into nude mice followed by treatment with or without cisplatin. Representative images of the nude mice are listed in Figure 4(a). The tumor growth curves are listed in Figure 4(b). Tumors weights were recorded and shown in Figure 4(c). The tumor weights were 0.77 ± 0.17 g in the untreated H1299 vec group, 0.45 ± 0.19 g in the cisplatin-treated H1299 vec group, 1.39 ± 0.15 g in the untreated H1299 PC4 group, and 1.18 ± 0.21 g in the cisplatin-treated H1299 PC4 group. As expected, the tumor weights were obviously reduced in the cisplatin-treated H1299 vec group in comparison with the cisplatin-treated H1299 PC4 group (*p* < 0.05). Interestingly, the tumor weights were also significantly reduced in the untreated H1299 vec group in comparison with the untreated H1299 PC4 group (*p* < 0.05). To more accurately reveal the impact of PC4 in decreasing LUAD cell sensitivity to CDDP, we further calculated the ratio of tumor weights. As shown in Figure 4(d), the average weight in the cisplatin-treated H1299 vec group was 58.81% of that in the untreated H1299 vec group, and the average weight in the cisplatin-treated H1299 PC4 group was 84.85% of that in the untreated H1299 PC4 group. Thus, cisplatin treatment led to a 41.19% reduction in tumor weight in the H1299 vec group compared to only a 15.15% reduction in the H1299 PC4 group (*p* < 0.05).

### 3.4. SOX9 Is a Downstream Target of PC4

**Figure 5:**
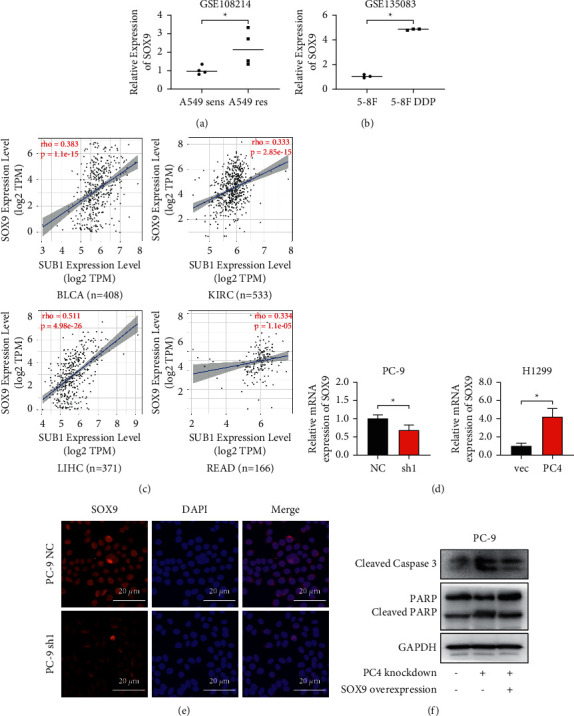
SOX9 acts as a downstream factor of PC4. (a), (b) The expression of SOX9 in the GEO datasets GSE108214 and GSE135083 was analyzed. (c) The correlation between PC4 and SOX9 in multiple cancer types was analyzed online. (d) qRT-PCR analysis revealed the relative SOX9 mRNA expression in PC4-knockdown and PC4-overexpression LUAD cells. (e) Immunofluorescence showed the alteration of SOX9 in PC4-knockdown PC-9 cells. (f) The expression of apoptotic proteins was analyzed after PC-9 PC4-knockdwon and SOX9-overexpression cells were treated with cisplatin. ^*∗*^*p* < 0.05.

GSE108214 analysis showed that SOX9 was elevated in the A549 res group compared to the A549 sens group (LogFC = 1.059, *p* = 0.028) (Figure 5(a)). Furthermore, in another GEO dataset, GSE135083, the SOX9 expression was also elevated in CDDP-resistant nasopharyngeal carcinoma 5-8F cells (5-8F DDP) compared to CDDP-sensitive 5-8F cells (5-8F) (LogFC = 3.807, *p* < 0.001) (Figure 5(b)). Next, the correlations between PC4 and SOX9 in multiple cancer types were analyzed online (http://timer.comp-genomics.org). As shown in Figure 5(c), PC4 and SOX9 were positively correlated in many types of cancers, including bladder urothelial carcinoma (BLCA) (*r* = 0.383, *p* < 0.001), kidney renal clear cell carcinoma (KIRC) (*r* = 0.333, *p* < 0.001), liver hepatocellular carcinoma (LIHC) (*r* = 0.511, *p* < 0.001), and rectum adenocarcinoma (READ) (*r* = 0.334, *p* < 0.001). Next, we wondered whether SOX9 acted as a downstream target of PC4. According to the qRT-PCR assay (Figure 5(d)), overexpression of PC4 obviously upregulated the expression of SOX9 mRNA (*p* = 0.004) in H1299 cells, while knockdown of PC4 inhibited SOX9 mRNA expression (*p* = 0.037) in PC-9 cells. Furthermore, the immunofluorescence results (Figure 5(e)) showed that knockdown of PC4 obviously inhibited the expression of SOX9 in PC-9 cells. To further validate whether SOX9 is a downstream factor of PC4 in mediating lung adenocarcinoma cell sensitivity to cisplatin, SOX9 was overexpressed in PC-9 PC4-knockdown cells and treated with cisplatin. As demonstrated in Figure 5(f), the caspase 3 and PARP cleavage activities were elevated in PC4-knockdown cells but decreased in PC4-knockdown and SOX9-overexpression cells. Overexpression of SOX9 obviously abrogated PC4 knockdown-induced cell apoptosis in PC-9 cells.

### 3.5. PC4 Is a Therapeutic Target of Multiple Drugs in Lung Adenocarcinoma Cells

**Figure 6:**
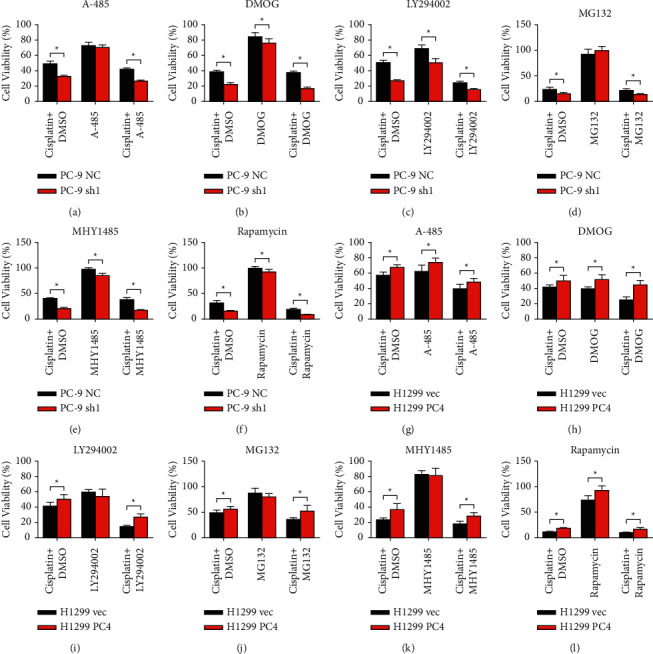
PC4 decreases lung adenocarcinoma cell sensitivity to DMOG and rapamycin. A-L PC4-knockdown or PC4-overexpression LUAD cells were treated with 1 *µ*M A-485, 1 mM DMOG, 30 *µ*M LY294002, 30 *µ*M MG132, 10 *µ*M MHY1485, or 30 nM rapamycin either alone or in combination with cisplatin. An individual cisplatin + DMSO control group was established for each drug. ^*∗*^*p* < 0.05.

Next, we evaluated whether PC4 participated in mediating lung adenocarcinoma cell sensitivity to other therapeutic drugs. The p300/CBP inhibitor A-485, the HIF-PH inhibitor DMOG, the PI3K/Akt pathway inhibitor LY294002, the proteasome inhibitor MG132, the mTOR activator MHY1485, and the mTOR inhibitor rapamycin were used in our research (Figures 6(a)–6(l)). As the CCK-8 tests discovered, the cell viability of the PC-9 NC and PC-9 sh1 cells was 72.83 ± 4.39% vs. 70.50 ± 3.39% (*p* > 0.05) after A-485 treatment, 84.67 ± 5.53% vs. 76.30 ± 5.83% (*p* < 0.05) after DMOG treatment, 68.71 ± 4.95% vs. 50.41 ± 5.29% (*p* < 0.05) after LY294002 treatment, 92.02 ± 10.40 vs. 99.60 ± 8.34% (*p* > 0.05) after MG132 treatment, 97.14 ± 3.03% vs. 85.27 ± 4.05% (*p* < 0.05) after MHY1485 treatment, and 99.02 ± 3.75% vs. 92.06 ± 4.78% (*p* < 0.05) after rapamycin treatment. The cell viability of the H1299 vec and H1299 PC4 cells was 62.18 ± 8.19% vs. 74.07 ± 5.61% (*p* < 0.05) after A-485 treatment, 39.60 ± 2.32% vs. 51.87 ± 6.16% (*p* < 0.05) after DMOG treatment, 59.48 ± 3.30% vs. 54.02 ± 9.51% (*p* > 0.05) after LY294002 treatment, 87.17 ± 9.95% vs. 79.95 ± 6.60% (*p* > 0.05) after MG132 treatment, 82.42 ± 5.09% vs. 80.95 ± 9.64% (*p* > 0.05) after MHY1485 treatment, and 73.10 ± 8.84% vs. 92.47 ± 8.56% (*p* < 0.05) after rapamycin treatment. These results showed that LUAD cell sensitivity to DMOG and rapamycin was significantly increased in the PC4-knockdown cells and decreased in the PC4-overexpression cells.

### 3.6. PC4 Destabilizes HIF-1*α* and Decreases the Synergetic Effects of DMOG and Cisplatin

**Figure 7:**
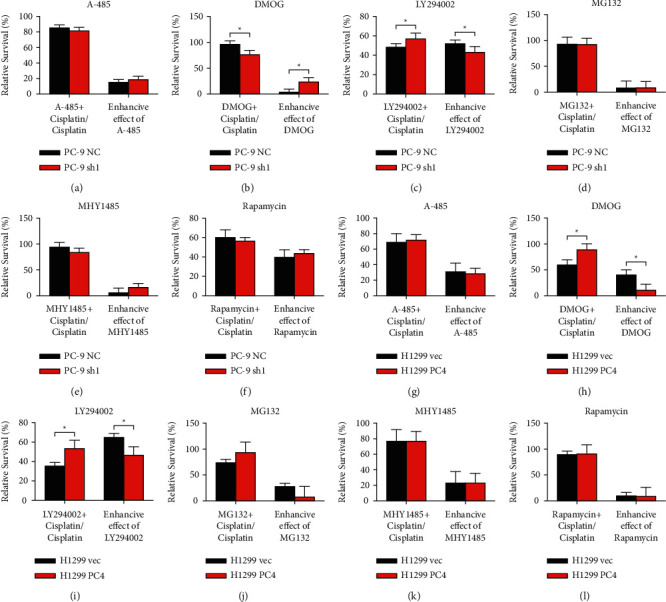
PC4 decreases the synergistic effect of DMOG and cisplatin. (a–l) The ratio of cell viability and relative enhancement of the effect of cisplatin were calculated. ^*∗*^*p* < 0.05.

Furthermore, we sought to evaluate the impact of PC4 in mediating the synergetic effects of cisplatin combined with other drugs. Thus, the cell viability ratios were calculated to evaluate the enhancive effect of each drug with cisplatin (Figures 7(a)–7(l)). A-485, MG132, MHY1485, and rapamycin showed no significant differences in enhancing the effects of cisplatin between the PC-9 NC and PC-9 sh1 cells. DMOG enhanced cisplatin's cytotoxic effects in PC-9 NC cells by 3.63 ± 6.39% compared to 23.74 ± 8.30% in PC-9 sh1 cells (*p* < 0.05). The enhancive effect of LY294002 on cisplatin was 51.88 ± 3.84% in PC-9 NC cells and only 43.00 ± 6.01% in PC-9 sh1 cells (*p* < 0.05). Likewise, A-485, MG132, MHY1485, and rapamycin possessed no significant differences in enhancing cisplatin's cytotoxic effect between the H1299 vec and H1299 PC4 cells (*p* > 0.05). The effect of DMOG on enhancing the effect of cisplatin was 40.33 ± 10.03% in the H1299 vec cells and 10.94 ± 11.63% in the H1299 PC4 cells (*p* < 0.05). The effect of LY294002 on enhancing the effect of cisplatin was 64.82 ± 4.04% in the H1299 vec cells and 46.62 ± 8.59% in H1299 PC4 cells (*p* < 0.05). Thus, overexpression of PC4 decreased the synergistic effect of DMOG and cisplatin while knockdown of PC4 increased it. Interestingly, the synergistic effect of LY294002 and cisplatin was inhibited in both the PC4-knockdown and PC4-overexpression LUAD cells.

**Figure 8:**
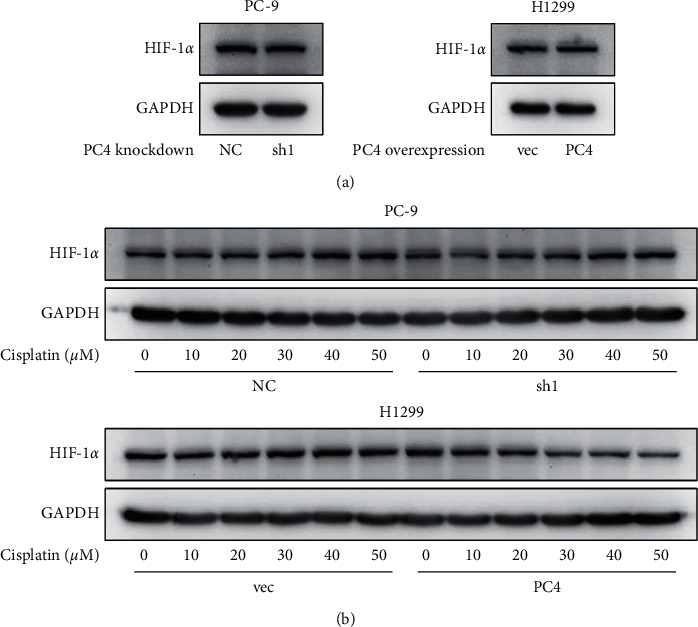
PC4 destabilizes HIF-1*α* upon cisplatin treatment. (a) The HIF-1*α* protein level was determined by western blot in PC4-knockdown and PC4-overexpression LUAD cells. (b) Increasing concentrations of cisplatin were added into PC4-knockdown and PC4-overexpression LUAD cells for 24 hours. The HIF-1*α* protein expression was evaluated by western blot. ^*∗*^*p* < 0.05.

As DMOG is an inhibitor of HIF-PH, which results in HIF-1*α* stabilization, we presume that the effect of PC4 in decreasing synergistic effect of DMOG and cisplatin is accompanied by changes in the expression of HIF-1*α*. First, the expression of HIF-1*α* in PC4-knockdown and PC4-overexpression cells was detected. However, as shown in Figure 8(a), neither knockdown nor overexpression of PC4 had any impact on the expression of HIF-1*α* in lung adenocarcinoma cells. Next, we assessed the impact of PC4 on the stability of HIF-1*α* upon cisplatin treatment. Increasing concentrations of cisplatin were administered to PC4-knockdown and PC4-overexpression cells for 24 hours. As shown in Figure 8(b), cisplatin in a concentration-dependent manner activated the expression of HIF-1*α* in both H1299 vec and PC-9 NC cells. Overexpression of PC4 decreased the stability of HIF-1*α* upon cisplatin treatment, while knockdown of PC4 further increased it.

## 4. Discussion

PC4 has been reported to participate in many cellular activities in cancers [13, 25], but its impact in mediating cell sensitivity to chemotherapeutic drugs remains to be elucidated. By analyzing the expression of PC4 in the GEO database, we discovered that PC4 was overexpressed in cisplatin-resistant LUAD A549 cells, indicating that PC4 may participate in the regulation of LUAD cell sensitivity to cisplatin.

Through lentivirus infection, PC4 was stably inhibited in PC-9 cells and overexpressed in H1299 cells. The *in vitro* study revealed the aggressive phenotype PC4 conferred to lung adenocarcinoma cells migration, which is consistent with previous research in other cancer cell types [13, 14]. Next, we discovered that PC4 decreased lung adenocarcinoma cell sensitivity to cisplatin *in vivo* and *in vitro*, as shown by a decreased cell viability, increased cell apoptosis, and lower tumor weight in PC4-knockdown group upon cisplatin treatment.

SOX9 is well studied in cancer chemotherapy, and its high expression and activation have been proven to promote cell resistance to chemotherapy in multiple types of cancers, including lung cancer [21, 26–28]. However, the relationship between PC4 and SOX9 remains determined. Based on previous findings, we wondered whether the role of PC4 in mediating lung adenocarcinoma sensitivity to cisplatin is associated with SOX9. First, we found that SOX9 was elevated in cisplatin-resistant lung adenocarcinoma A549 cells (GSE108214) and nasopharyngeal carcinoma 5-8F cells (GSE135083) through GEO dataset analysis. Next, we revealed positive correlations between PC4 and SOX9 in multiple cancers. The qRT-PCR results further demonstrated that knockdown of PC4 downregulated the SOX9 mRNA level, while overexpression of PC4 upregulated it. Immunofluorescence showed that PC4 knockdown obviously inhibited the expression of SOX9. These results implicated that SOX9 acts as a downstream factor of PC4. Finally, overexpression of SOX9 abrogated the apoptosis induced by PC4 knockdown in lung adenocarcinoma cells, suggesting that SOX9 acts as a downstream factor of PC4 in mediating cell sensitivity to cisplatin.

Previous CCK-8 assays revealed that PC4 knockdown or overexpression had no impact on the growth of PC-9 and H1299 cells. However, treatment with cisplatin induced significant changes in the OD values, which was due to the changes in PC4 expression and was a direct reflection of the impact of PC4 in decreasing LUAD cell sensitivity to cisplatin. Based on this, CCK-8 tests were used to detect the cell response to more therapeutic drugs and their synergistic effect with cisplatin to determine whether PC4 also mediated cell sensitivity. As a universal solvent, DMSO is widely used to dissolve multiple small molecules. However, previous research demonstrated that DMSO interacted with platinum complexes, decreasing their cytotoxic effects [29]. The volume of DMSO had direct impacts on the cytotoxic effects of cisplatin. In our CCK-8 tests, we used multiple other drugs, which were all dissolved in DMSO, in combination with cisplatin. As the volume for each drug added to the cells was different, to eliminate the impact of DMSO on the accuracy of our tests, we set an individual cisplatin + DMSO control group for each drug, replacing the drug with the same volume of DMSO. The results revealed that the HIF-PH inhibitor DMOG and the mTOR inhibitor rapamycin mediated significant changes in cell viability in both PC4-knockdown and PC4-overexpression cells. DMOG and rapamycin induced significantly lower cell viability in PC4-knockdown cells and higher cell viability in PC4-overexpression cells, indicating that PC4 also participates in mediating cell sensitivity to DMOG and rapamycin.

To accurately evaluate the synergistic effect of other drugs on cisplatin, we first calculated the ratio of cell viability (cell viability _combinational therapy_/cell viability _cisplatin_ × 100%). Then, the effect of each drug on enhancing the effect of cisplatin was calculated as (1- cell viability _combinational therapy_/cell viability _cisplatin_) × 100%. The results showed that DMOG exerts synergistic enhancive effects on cisplatin, which were altered by PC4 knockdown and overexpression. DMOG synergistically enhanced cytotoxic effect of cisplatin in PC-9 and H1299 cells, and this effect was enhanced upon PC4 knockdown and reduced upon PC4 overexpression. As DMOG is an inhibitor of HIF-PH, which results in HIF-1*α* stabilization and accumulation, we further investigated whether PC4 impacted the expression of HIF-1*α*. Surprisingly, HIF-1*α* was not altered by PC4. Next, we evaluated the stability of HIF-1*α* upon cisplatin treatment and found that cisplatin stabilized HIF-1*α* in both PC-9 and H1299 cells, and overexpression of PC4 decreased the stability of HIF-1*α* upon cisplatin treatment, while knockdown of PC4 increased it. These results suggested that the PC4-induced decrease in lung adenocarcinoma cell sensitivity to cisplatin is associated with a decrease in HIF-1*α* stability.

In summary, our research showed that PC4 participates in mediating LUAD cell sensitivity to cisplatin, DMOG, and rapamycin and enhances the synergistic effect of DMOG and cisplatin. Mechanistically, PC4 governs multiple downstream pathways associated with chemotherapy resistance, including the SOX9 and HIF-1*α* pathways. Thus, PC4 is a promising chemotherapeutic target in LUAD.

## Data Availability

Upon reasonable request, relative data could be obtained from the corresponding author.
